# Poor-Prognosis Patients Affected by Glioblastoma: Retrospective Study of Hypofractionated Radiotherapy with Simultaneous Integrated Boost and Concurrent/Adjuvant Temozolomide

**DOI:** 10.3390/jpm11111145

**Published:** 2021-11-04

**Authors:** Fabiana Gregucci, Alessia Surgo, Ilaria Bonaparte, Letizia Laera, Maria Paola Ciliberti, Roberta Carbonara, Maria Annunziata Gentile, David Giraldi, Roberto Calbi, Morena Caliandro, Nicola Sasso, Salvatore D’Oria, Carlo Somma, Gaetano Martinelli, Giammarco Surico, Giuseppe Lombardi, Alba Fiorentino

**Affiliations:** 1 Department of Radiation Oncology, Miulli General Regional Hospital, 70021 Acquaviva delle Fonti, Bari, Italy; fabianagregucci@gmail.com (F.G.); surgo.alessia@gmail.com (A.S.); ilariabonaparte@libero.it (I.B.); m.ciliberti@miulli.it (M.P.C.); roberta.carbonara@yahoo.it (R.C.); morenacaliandro@yahoo.it (M.C.); 2 Department of Medical Oncology, Miulli General Regional Hospital, 70021 Acquaviva delle Fonti, Bari, Italy; l.laera@miulli.it (L.L.); n.sasso@miulli.it (N.S.); giammarco.surico@miulli.it (G.S.); 3 Department of Radiology, Miulli General Regional Hospital, 70021 Acquaviva delle Fonti, Bari, Italy; m.gentile@miulli.it (M.A.G.); r.calbi@miuli.it (R.C.); g.martinelli@miulli.it (G.M.); 4 Department of Neurosurgery, Miulli General Regional Hospital, 70021 Acquaviva delle Fonti, Bari, Italy; d.giraldi@miulli.it (D.G.); s.doria@miulli.it (S.D.); c.somma@miulli.it (C.S.); 5 Department of Oncology, Oncology 1, Veneto Institute of Oncology IOV-IRCCS, 35128 Padua, Padova, Italy; giuseppe.lombardi@iov.veneto.it

**Keywords:** glioblastoma, poor prognosis, radiotherapy, chemotherapy

## Abstract

Background: Glioblastoma (GBM) is a very poor-prognosis brain tumor. To date, maximal excision followed by radiochemotherapy, in 30 fractions, is the standard approach. Limited data are present in the literature about hypofractionated radiotherapy (hypo-RT) in GBM poor prognosis patients. Thus, this retrospective study was conducted to evaluate efficacy and toxicity of hypo-RT with simultaneous integrated boost (SIB) in association with temozolomide (TMZ) in this patient setting. Methods: Poor-prognosis GBM patients underwent surgery (complete, subtotal or biopsy) followed by SIB-hypo-RT and concomitant/adjuvant TMZ. The prescription dose was 40.05 Gy (15 fractions) with a SIB of 52.5 Gy (3.5 Gy/fraction) on surgical cavity/residual/macroscopic disease. Volumetric modulated arc therapy was performed. Results: From July 2019 to July 2021, 30 poor-prognosis patients affected by GBM were treated by SIB-hypo-RT; 25 were evaluated in the present analysis due to a minimum follow up of 6 months. The median age and KPS were 65 years and 60%, respectively. At the median follow-up time of 15 months (range 7–24), median and 1-year overall survival and progression-free survival were 13 months and 54%, and 8.4 months and 23%, respectively. No acute or late neurological side effects of grade ≥ 2 were reported. Grade 3–4 hematologic toxicity occurred in three cases. Conclusion: SIB-hypo-RT associated with TMZ in poor-prognosis patients affected by GBM is an effective and safe treatment. Prospective studies could be warranted.

## 1. Introduction

Glioblastoma (GBM) is the most common and devastating malignancy of the brain with a median survival time of 12–18 months [1]. Maximal safe resection, when feasible, is the first effective treatment, followed by adjuvant radiotherapy (RT) and chemotherapy with temozolomide [1]. Radical debulking, tumor size, performance status and the patient’s age are related to overall survival (OS) [1]. 

The first data supporting the use of adjuvant RT, alone or in association with bis-chloroethyl nitrosourea (BCNU), to increase OS and progression free survival (PFS) were reported more than 40 years ago [2]. In 2005, the introduction of concurrent and adjuvant temozolomide (TMZ) with RT (60 Gy in 30 fractions), adopted by the European Organization for Research and Treatment of Cancer (EORTC) and the National Cancer Institute of Canada (NCIC) in a Phase III randomized trial, showed an improvement in terms of oncological outcome. In fact, the 2-year OS and PFS rates were 27% and 10.7%, respectively [1]. Considering the high incidence of local recurrence in GBM, generally within 2 cm from the original edges of the tumors, and the patients’ death due to local progression, the goal of the treatment approach should be the improvement of local control [3,4,5]. Therefore, postoperative RT modalities, including total dose and new fractionation schedules, have been evaluated for these patients [3,4,5]. 

The technological improvement in RT, including the introduction of intensity modulated radiotherapy (IMRT/VMAT) and image-guided radiotherapy (IGRT), with more precise target coverage sparing the surrounding healthy tissue, has led to hypofractionated (hypo-RT) schemes [6,7]. In fact, several hypo-RT studies in high-grade gliomas have reported good results in terms of efficacy and toxicity [8,9,10,11,12,13]. Malmstrom et al. [12] in 2012 published a randomized Phase III trial with three comparison arms: TMZ alone vs. standard RT (60 Gy in 30 fractions) vs. hypo-RT (34 Gy in 10 fractions) in patients older than 60 years. The authors concluded that in elderly patients over 70 years, both TMZ and hypo-RT should be considered as standard treatment options. The multicentric Phase II study published by Scoccianti et al. [13] evaluated the OS, PFS and toxicity of a hypo-RT with simultaneous integrated boost (SIB) in association with TMZ in patients with relatively good prognosis (RPA Classes III–IV). All patients received 52.5 Gy in 15 fractions and, concomitantly, 67.5 Gy to the SIB volume. Despite low accrual, the authors concluded that hypo-RT is a reasonable and feasible option for GBM patients [13]. 

Few data are present in literature about hypo-RT and poor-prognosis patients. Moreover, the definition of poor-prognosis GBM has not been defined, due to the different pathological and clinical features. In the setting of poor-prognosis patients, no standard of care is available, suggesting that RT alone, TMZ alone or best supportive care could be proposed. However, in this setting, several results were correlated only with elderly and frail people, for whom the reduction of RT fractions could represent a valid option with a median OS of 6–8 months [11,14,15,16,17,18,19,20,21,22,23,24,25,26,27]. 

For poor-prognosis patients other than those with advanced age and frailty, the data are sparse.

Based on this background and the lack of specific data in this setting, aim of the present retrospective study was to assess the toxicity profile and outcome in poor-prognosis patients affected by GBM treated with hypo-RT with SIB and concomitant/adjuvant TMZ. Moreover, a literature review on the management of elderly and/or frail patients affected by high-grade gliomas treated with hypo-RT was conducted.

## 2. Materials and Methods

Inclusion criteria were as follows: newly diagnosed with histologically proven GBM; poor-prognosis patients; more than 18 years of age; Karnofsky performance scale (KPS) more than 50; all patients belonged to recursive partitioning analysis (RPA) [28] Classes IV, V and VI (IV: age < 50 years, KPS < 90; age ≥ 50 years, KPS ≥ 70, total or subtotal resection, good neurological function; V: age ≥ 50 years, KPS < 70, stereotactic biopsy, GBM, neurological function that inhibits the ability to work; VI: age ≥ 50 years, KPS < 70, abnormal mental status); patient’s refusal to receive 30 fractions; and adequate bone marrow, renal and liver function. All patients underwent debulking surgery; in the case of tumor unresectability, a biopsy procedure was performed in order to obtain the tumor diagnosis. Subtotal resection was defined as less than 100% and more than 50% of the tumor. The exclusion criteria were prior brain radiotherapy, brainstem tumors, age below 18 years; KPS below 50. Informed consent was obtained from all patients included in the study.

The definition of poor prognostic factors other than advanced age included KPS or RPA class, neurological symptoms after surgical procedures or symptoms of mass effect, high tumor burden, unresectable or multifocal lesions, high comorbidity and potential low treatment compliance due to rapidly progressive disease.

The end points of the study were the evaluation of PFS, OS, acute and late toxicity. 

**Figure 1:**
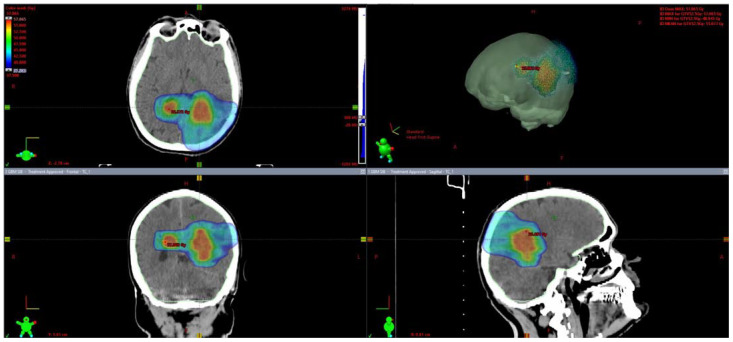
Example of a volumetric arc therapy treatment plan with simultaneous integrated boost.

Patients were immobilized in the supine position with a thermoplastic open mask (Solstice^TM^ SRS Immobilization System, CIVCO^®^ Radiotherapy). Computed tomography (CT) simulation was performed without contrast, acquiring slices of 1 mm thickness. Co-registration with post-surgery magnetic resonance imaging (MRI) was mandatory. Organs at risk (OARs) were contoured: brain (normal brain minus planning target volume (PTV)), eyes, lens, optic chiasm, optic nerves, brainstem and spinal cord, and hippocampus. The gross tumor volume (GTV) was defined on the T1 with gadolinium-weighted MRI, and it included the surgical cavity with or without any residual contrast-enhanced lesion or the entire lesion, if only a biopsy was performed. The clinical target volume for the SIB (CTV1) was defined as the GTV without the margin, whereas the clinical target volume for the lower-dose volume (CTV2) was obtained by adding a 10–15 mm margin to the CTV1, respecting the anatomical barriers and OARs. The PTV1 and PTV2 were created by a CTV expansion of 2–3 mm. The total dose was 52.5 Gy for PTV1 and 40.05 Gy for PTV2 in 15 fractions. An example is shown in Figure 1. 

For RT planning, 6× flattening filter free (FFF) and volumetric modulated arc therapy (VMAT) plans were generated with two or more coplanar or non-coplanar partial arcs by TrueBeam^TM^ (Varian Medical System). All treatment plans were optimized for PTV so that more than 98% of the PTV received at least 95% of the prescribed dose. The prescribed PTV1 dose of 52.5 Gy in 3 weeks corresponded to a biological effective dose (BED) similar to standard RT (BED_10_ of 70.88 Gy versus 72 Gy, respectively) [13,17]. 

During RT, IGRT with cone-beam CT (CBCT) and real-time surface-guided RT, using AlignRT^®^, was performed daily prior to and during the RT session. Concomitant TMZ at a dose of 75 mg per square meter of body-surface area per day for 21 consecutive days, 7 days/week, was administered from Day 1 until the final day of hypo-RT [1]. Blood count was monitored weekly throughout the treatment. During hypo-RT, patients received 2–4 mg/day of dexamethasone with a proton pump inhibitor. If feasible, patients underwent 6–12 cycles or until progression of adjuvant TMZ (150–200 mg/mq/day, 5 days every 28 days) 4 weeks from the end of RT [1]. During treatment, antiepileptic and antiemetic drugs were administered when necessary. 

Clinical evaluations and MRI were performed 45–60 days after the end of the hypo-RT, then every 2–3 months for the first 2 years or as appropriate. At each visit, neurological status and the severity of complications were rated according to the National Cancer Institute Common Toxicity Criteria (NCI-CTC Version 4). Adverse neurological events were considered to be consequences of treatment in the absence of disease progression. The RANO response criteria were adopted to evaluate the disease status [29].

The outcome variables were acute and late toxicity, PFS and OS. The acute and late toxicity were considered as categorial variables, defined according to the NCI-CTC scale. Moreover, the presence/absence of radio-necrosis was recorded as a dichotomous variable. The PFS was calculated from the date of RT to the time of progression or the last follow-up date. The OS was calculated from the date of RT to death for any cause or the last follow-up date. The Kaplan–Meier method was used to evaluate PFS and OS. A log-rank test was used to compare the different subgroups in univariate analysis. Multivariate analysis was performed to determine the independent prognostic factors by using the Cox regression model. A two-sided *p*-value equal to or less than 0.05 was considered statistically significant. The following prognostic factors were evaluated: sex, age (<65 vs. ≥65), KPS (≤60 vs. >60), RPA (IV vs. V + VI), CCI (<8 vs. ≥8), mass effect (yes vs. not), multifocal tumor (yes vs. no), surgery (surgery vs. biopsy), resection (complete vs. incomplete), O6-methylguanin-DNA-methyltransferase (MGMT) promoter methylation status (methylated vs. not methylated), GTV (≤50 vs. >50) and PTV (≤200 vs. >200). Data management and statistical analysis were conducted using the open-source R platform (version 3.5.2). 

## 3. Results

From July 2019 to July 2021, 30 poor-prognosis patients affected by GBM underwent hypo-RT with SIB. Of these, 25 cases were evaluated in the present analysis because they had a minimum follow up of 6 months. 

Table 1 reports the clinical characteristics of the study population. The median age, KPS and Charlson comorbidity index (CCI) were 65 years (range 37–82), 60% (range 50–90%) and 8 (range 5–14), respectively. Eighteen patients (72%) had RPA Class ≥V. All patients were affected by wild-type GBM isocitrate dehydrogenase (IDH)1; in 76% of cases, MGMT was unmethylated. Subtotal resection was performed in 22 patients (88%), including 10 cases (40%) of unresectable disease in which biopsy alone was performed. At diagnosis, 15 patients (60%) showed signs or symptoms of mass effect and required anti-edema therapy. The median time between the surgical procedure and RT was 8 weeks (range 2–14). 

Compliance with SIB hypo-RT with TMZ was 100%. 

Adjuvant TMZ was administered to 20 (80%) patients (six cycles median, range 3–15), while five patients were not eligible due to their clinical condition and progression of the disease. 

**Figure 2:**
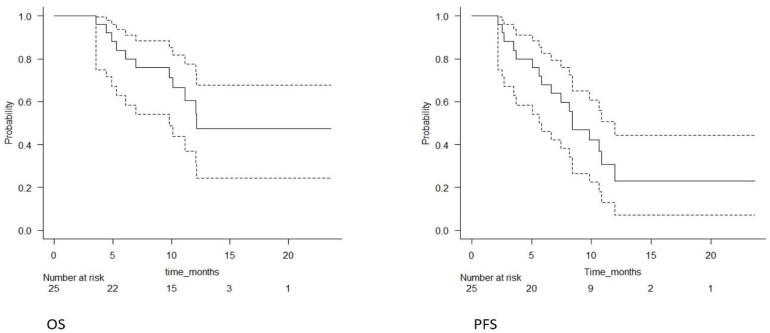
Overall survival and progression-free survival of the entire population (solid lines); confidence intervals (dotted lines).

At the 15 month median follow-up, 14 patients (56%) were alive: three (12%) showed a partial treatment response, three (12%) had a stable disease and eight (32%) showed progression of the disease. The median OS was 13 months (95% CI 9.8–na) and 1-year OS was 54% (95% CI 31–73%); the median PFS was 8.4 months (95% CI 5.8–11.9) and the 1-year PFS was 23% (95% CI 7–44%). The OS and PFS Kaplan–Meyer survival curves are shown in Figure 2. 

Regarding toxicity, no acute or late neurological side effects of more than Grade 2 were reported, without cases of radio-necrosis. Grade 3–4 hematologic toxicity occurred in three cases. 

Neurocognitive assessments pre- and post-hypo-RT were available in a limited number of patients (15%) and are therefore unreliable. 

Of the 17 cases of disease progression, eight received re-irradiation followed by second-line systemic therapy (regorafenib or fotemustine), three received regorafenib alone and six were evaluated for best supportive care.

### Prognostic Factors for OS and PFS

The univariate and multivariate prognostic factors influencing OS are shown in Table 2. Seven significant variables in the univariate analysis (age, RPA, multifocal tumor, resection, MGMT status, GTV and PTV) were entered into the multivariable model. As a result, MGMT non-methylation (HR: 0.61, 95% CI: 0.02–13.66, *p* = 0.05), GTV > 50 cc (HR: 4.83, 95% CI: 0.83–27.88, *p* = 0.01) and PTV > 200 cc (HR:2.14, 95% CI: 0.01–9.87, *p* = 0.02) were significant negative prognostic factors for survival. The univariate and multivariate prognostic factors influencing PFS are shown in Table 3. Six significant variables in the univariate analysis (mass effect, multifocal tumor, surgery, resection, MGMT status and PTV) were entered into the multivariable model. As a result, multifocal tumor (HR: 1.76, 95% CI: 0.31–8.12, *p* = 0.05), incomplete resection (HR: 2.91, 95% CI: 0.31–27.07, *p* = 0.01), MGMT unmethylation (HR: 0.79, 95% CI: 0.11–6.25, *p* = 0.03) and PTV >200 cc (HR: 1.99, 95% CI: 0.26–5.37, *p* = 0.05) were significant negative prognostic factors for disease progression.

## 4. Discussion

To our knowledge, there are limited data regarding the role of hypo-RT plus TMN in poor-prognosis GBM patients, other than advanced age. 

Older age, clinical performance status and RPA class are the most relevant prognostic factors; due to the rapid progression of the disease, up to 10% of these patients discontinue or do not start treatment [17,18]. Based on this later consideration, the possibility of reducing the overall radiotherapy treatment time without detrimental effects on the outcomes, represent the real challenge for this subgroup of patients.

In 1994, Bauman et al. showed that a palliative RT course of 30 Gy in 10 fractions could be useful in terms of survival in elderly GBM patients with a low pretreatment KPS (<50) [27]. A Phase III trial conducted by Roa and colleagues compared two different hypofractionation schemes (40 Gy in 15 fractions and 25 Gy in five fractions) without concurrent TMZ in patients over 65 years of age with KPS > 50 [11]. However, the optimal dose in this subpopulation remained unclear. No differences in OS, PFS or quality of life were observed between the two arms. However, this study has been criticized for its low statistical relevance and the presence of other bias (differences in the patients’ characteristics, trial design and treatment delivery). On the other hand, the Nordic Clinical Brain Tumor Study reported poorer OS outcomes in elderly GBM patients treated with standard RT (60 Gy in 30 fractions) in comparison with those who received hypo-RT (34 Gy in 10 fractions) [12]. Pedretti et al. published a randomized trial in which 14 poor-prognosis patients were enrolled to received hypo-RT (30 Gy in six fractions) and 17 received TMZ exclusively (TMZ 200 mg/m^2^/day for 5 days every 28 days). RPA VI (*p* = 0.048) and the absence of MGMT methylation (*p* = 0.001) worsened OS significantly. Biopsy (*p* = 0.048), RPA Class VI (*p* = 0.04) and TMZ (*p* = 0.007) worsened PFS. Despite the limited accrual, the authors concluded that RT had a better PFS, without significant differences in OS [24].

However, despite the limitations highlighted, short-course RT and standard RT were equivalent in terms of outcome and safety [7,8,9,10,11,12,13,14,15,16,17,18,19,20,21,22,23,24,25,26,27]. These results led to an increased use of hypo-RT schemes in frail and elderly GBM patients. 

However, few data have been produced for GBM patients with poor prognostic factors such as high tumor burden, unresectable or multifocal lesions, low Karnofsky performance status (KPS) or the presence of significant comorbidities [15,17].

For this reason, the present analysis reported data regarding the use of hypo-RT with concomitant and adjuvant TMZ in GBM patients with poor prognosis factors other than age. The present population, in fact, had a median age of 65 years, all patients had a high comorbidity score; 40% of the patients received only a biopsy, around 30% of patients had multifocal disease, more than 50% of patients had a high disease burden (PTV more than 200 cc), 80% had unmethylated MGMT and 72% of cases were in RPA Class V–VI. 

This analysis is the first report about the use of a concomitant boost for poor-prognosis GBM patients, showing the safety and the efficacy of the approach. As shown in Table 4, overall survival in this patient setting ranged from 5 to 9 months. Thus, the present results are interesting, due to the median OS and PFS of 13 and 8 months.

Regarding poor prognosis, our analysis could be compared with two published papers, due to the similar hypo-RT schedule and enrolled population [15,17]. 

The Phase II trial published by Navarria et al. showed the feasibility and effectiveness of a hypo-RT schedule with adjuvant TMZ in 30 poor-prognosis GBM patients [17]. The authors prescribed a total dose of 52.5 Gy in 3 weeks. The aim of the study was to improve the median OS from 6 to 12 months, but this was not achieved; moreover, 10% of the patients did not complete the planned hypo-RT. The median PFS and 12-month PFS rates were 5 months and 20.0%, while median and 12-month OS rates of 8 months and 30%, respectively, were achieved [17]. Ten percent of patients experienced acute or late neurologic toxicity.

Jablonska et al. proposed hypo-RT (40 Gy in 15 fraction) with concurrent TMZ in 17 GBM patients with poor prognostic factors (elderly age, post-surgical neurological complications, high tumor burden, unresectable or multifocal lesions) [15]. The authors showed excellent compliance, with a low toxicity profile and a PFS of 60% at 6 months, 33% at 1 year and 13% at 2 years; while the median OS time was 7 months; the 6-month, 1-, and 2-year OS rates were 62%, 46%, and 18%, respectively [15]. 

Regarding the hypo-RT schedule, the present study is the first one reporting a SIB-hypo-RT schedule for poor-prognosis patients: 40 Gy in 15 fractions for PTV (similar to Jablonska et al.’s study [15]) and 52.5 Gy simultaneously to residual mass (similar to Navarria’s trial [17]). Based on the present schedule, all patients completed the planned treatment and, probably due to the boost dose to residual mass, the median PFS was high (8.4 months). Our data reported a low toxicity rate and a high median survival compared with the other reports (7 vs. 8 vs. 13 months), probably due to the SIB dose and second-line therapy at recurrence. In fact, among 17 recurrences, eight patients received a second irradiation with or without second-line chemotherapy such as regorafenib [30,31,32].

The most important limitations of the present analysis are the poor sample size and the retrospective nature of the study. However, in GBM patients with poor prognostic factors other than age, the two studies published in the literature by Navarria and Jablonska enrolled 30 and 17 patients, respectively, due to the difficulty of enrolling this type of patients. In fact, in real-world data, patients with primary GBM, poor performance status (KPS < 60) and age > 70, multifocal disease, high comorbidities correlated with a high risk of mortality [33,34,35] and other negative prognostic factors are candidates for TMZ or best supportive care (BSC).

Moreover, due to the latter motivation, it will be difficult to collect strong data in terms of prospective or randomized trials for this population.

## 5. Conclusions

The present results suggest that the use of SIB hypo-RT (52.5/40 Gy in 15 fractions) with concurrent and adjuvant TMZ could also be contemplated in poor-prognosis GBM patients. However, a direct comparison among BSC, TMZ alone and hypo-RT is required in order to evaluate the best strategy to improve the outcome and quality of life.

## Figures and Tables

**Table 1:** Patients and tumor characteristics.

**Number of Patients**		25	
**Sex**			
	Male	18	(72%)
	Female	7	(28%)
**Age**			
	Median (range) in years	65	(37–82)
	<60 years	5	(20%)
	60–65 years	11	(44%)
	>65 years	9	(36%)
**KPS Score**			
	Median (range), in %	60	(50–90)
	<60%	5	(20%)
	60–70%	16	(64%)
	>70%	4	(16%)
**RPA Class**			
	Median (range)	V	(IV–VI)
	IV	7	(28%)
	V	10	(40%)
	VI	8	(32%)
**CCI**			
	Median (range)	8	(5–14)
	<7	6	(24%)
	7–9	18	(72%)
	>9	2	(4%)
**Mass Effect**			
	Yes	15	(60%)
	No	10	(40%)
**Surgery**			
	Complete	3	(12%)
	Incomplete	12	(48%)
	Unresectable (biopsy)	10	(40%)
**Multifocal Tumor**			
	Yes	7	(28%)
	No	18	(72%)
**Poor Molecular Factors**			
	wild-type IDH	25	(100%)
	Unmethylated MGMT	19	(76%)
**Gross Tumor Volume**			
	Median (range), in cc	50	(31–135)
	≤50 cc	16	(64%)
	>50 cc	9	(36%)
**Planning Target Volume**			
	Median (range), in cc	220	(117–358)
	≤200 cc	12	(48%)
	>200 cc	13	(52%)

**Table 2:** Univariate and multivariate analysis of prognostic factors for overall survival.

Variable	Univariate			Multivariate		
	HR	95%CI	*p*	HR	95%CI	*p*
Sex	1.23	0.3–2.3	0.7	-	-	-
Age (≥65 years)	1.622	0.48–5.38	0.04	2.88	0.72–11.43	0.1
KPS (≤60%)	0.42	0.05–3.32	0.4	-	-	-
RPA (≥ V)	1.78	0.37–8.38	0.04	0.19	0.01–2.78	0.2
CCI (≥8)	1.201	0.34–4.17	0.7	-	-	-
Mass effect (yes)	1.51	0.39–5.81	0.5	-	-	-
Multifocal tumor (yes)	1.92	0.56–6.61	0.02	2.95	0.51–16.98	0.2
Surgery (yes)	0.82	0.24–2.73	0.7	-	-	-
Resection (incomplete)	2.29	0.04–6.43	0.01	0.96	0.16–5.78	0.9
MGMT methylation (absent)	0.25	0.03–2.02	0.04	0.61	0.02–13.66	0.05
GTV (>50 cc)	5.208	1.37–19.71	0.01	4.83	0.83–27.88	0.01
PTV (>200 cc)	0.29	0.08–1.06	0.06	2.14	0.01–9.87	0.02

**Table 3:** Univariate and multivariate analysis of prognostic factors for progression-free survival.

Variable	Univariate			Multivariate		
	HR	95% CI	*p*	HR	95% CI	*p*
Sex	0.81	0.27–2.39	0.7	-	-	-
Age (≥65 years)	0.77	0.28–2.14	0.6	-	-	-
KPS (≤60%)	0.89	0.25–3.17	0.8	-	-	-
RPA (≥ V)	0.83	0.28–2.48	0.7	-	-	-
CCI (≥8)	0.95	0.35–2.54	0.9	-	-	-
Mass effect (yes)	1.47	0.05–4.23	0.04	1.32	0.29–5.97	0.7
Multifocal tumor (yes)	1.38	0.04–6.72	0.04	1.76	0.31–8.12	0.05
Surgery (yes)	1.76	0.02–3.04	0.05	1.32	o.18–3.27	0.7
Resection (incomplete)	2.96	0.3–22.42	0.02	2.91	0.31–27.07	0.01
MGMT methylation (absent)	1.99	0.04–4.06	0.06	0.79	0.11–6.25	0.03
GTV (>50 cc)	0.82	0.31–2.24	0.7	-	-	-
PTV (>200 cc)	1.95	0.04–3.74	0.06	1.99	0.26–5.37	0.05

**Table 4:** Literature review of studies regarding elderly/frail patients with high-grade gliomas and treated with hypofractionated RT.

Author/Publication Year	Study Years	Study Type	Patient Selection	Comparison	No. of Patients	RT Schedule	Median PFS	Median OS	Toxicities
Phillips et al. 2003 [9]	1990–1996	Randomized Phase III	Age > 45 y ECOG 0–3	Hypo-RT Standard RT	32 36	35 Gy/10 fx (WB) 60 Gy/30 fx	NS	8.7 months 10.3 months	None
Roa et al. 2004 [11]	1996–2001	Randomized Phase III	Age ≥ 60 y KPS ≥ 50	Hypo-RT Standard RT	48 47	40 Gy/15 fx 60 Gy/30 fx	NS	5.6 months 5.1 months	NS
Malmström et al. 2012 [12]	2000–2009	Randomized Phase III	Age ≥ 60 y ECOG 0–2	TMZ Hypo-RT Standard RT	93 98 100	NA 34 Gy/10 fx 60 Gy/30 fx	NS	8.3 months 7.5 months 6 months	No G > 3 acute toxicity
Roa et al. 2015 [18]	2010–2013	Randomized Phase III	Age ≥ 65 y KPS 50–70	Hypo-RT Hypo-RT	48 50	25 Gy/5 fx 40 Gy/15 fx	4.2 months 4.2 months	7.9 months 6.4 months	No G > 3 acute toxicity
Guedes de Castro et al. 2017 [19]	NS	Randomized Phase III	Age ≥ 65 y KPS 50–70	Hypo-RT Hypo-RT	26 35	25 Gy/5 fx 40 Gy/15 fx	4.3 months 3.2 months	6.8 months 6.2 months	No G > 3 acute toxicity
Perry et al. 2017 [16]	2007–2013	Randomized Phase III	Age ≥ 65 y ECOG 0–2	Hypo-RT + TMZ Hypo-RT	281 281	40 Gy/15 fx	5.3 months 3.9 months	9.3 months 7.6 months	No G > 3 acute toxicity
Pedretti et al. 2019 [24]	2010–2015	Randomized Phase II	RPA Class 5 or 6	Hypo-RT alone TMZ alone	14 17	30 Gy/6 fx over 2 weeks	3.8 months	6.3 months	No G > 3 acute toxicity
Bauman et al. 1994 [27]	1990–1992	Prospective	Age ≥ 65 y KPS ≤ 50	Hypo-RT	29	30 Gy/10 fx (WB)	NS	6 months	NS
Thomas et al. 1994 [29]	1991–1993	Prospective	KPS ≤ 50 or Age 55–70 y KPS 50–70 or Age ≥ 70 y	Hypo-RT	38	30 Gy/6 fx over 2 weeks	NS	6 months	None
Hulshof et al. 2000 [19]	1988–1998	Prospective	Age ≥ 65 y MRC ≥ 2	Hypo-RT Hypo-RT Standard RT	48 41 66	28 Gy/4 fx 40 Gy/8 fx 66 Gy/33 fx	NS	6.6 months 5.6 months 7 months	Mild; No difference between groups
Minniti et al. 2009 [21]	2002–2006	Prospective	Age ≥ 70 y KPS ≥ 60	Hypo-RT + adj TMZ	43	30 Gy/6 fx over 2 weeks	6.3 months	9.3 months	8 patients presented neurological deterioration (Grade 2/3 confusion and/or somnolence). 12 patients had Grade 3/4 hematological toxic effects
Omuro et al. 2014 [26]	NS	Prospective	Age ≥ 18 years (median 55 y) KPS ≥ 70 (median 90) Partial resection or biopsy (75%)	Hypo-RT + TMZ + BEV	40	30 Gy/6 fx over 2 weeks	10 months	19 months	None
Navarria et al. 2019 [17]	2013–2016	Prospective	Age ≥ 70 y KPS ≤ 60	Hypo-RT	30	52.5 Gy/15 fx	5 months	8 months	No severe acute or late neurologic toxicity was recorded
McAleese et al. 2003 [8]	1991–1999	Retrospective	KPS ≤ 50 or Age 50–70 y KPS 50–90 or Age ≥ 70 y	Hypo-RT	92	30 Gy/6 fx over 2 weeks	NS	5 months	NS
Chang et al. 2003 [10]	1988–2001	Retrospective	RPA Class ≥ 4	Hypo-RT	59	50 Gy/20 fx	3.9 months	7 months	3 patients showed radio-necrosis
Minniti el al. 2015 [23]	2004–2013	Retrospective	Age ≥ 65 y KPS ≥ 60	Hypo-RT + TMZ Standard RT + TMZ	116 127	40 Gy/15 fx 59.4–60 Gy/30–33 fx	6.7 months 5.6 months	12.5 months 12 months	28 patients receiving standard RT and 11 subjected to short-course RT had acute worsening of neurologic status. 20 patients receiving standard RT and 3 patients receiving short-course RT had late neurologic deterioration (G2–3 cognitive disability) G3–4 thrombocytopenia and lymphocytopenia were seen in 24 patients and 51 patients. G3 neutropenia developed in 14 patients, and 10 patients displayed G3 anemia
Jablonska et al. 2019 [15]	2010–2017	Retrospective	RPA Class ≥ 4	Hypo-RT with SIB + TMZ	17	50–45–40 Gy/15 fx	7 months	7 months	No acute G3–5 toxicities were observed. Radio-necrosis occurred in 1 patient.
Present study	2019–2021	Retrospective	Poor prognosis RPA Class ≥ 4	Hypo-RT with SIB + TMZ	25	52.5–40 Gy/15 fx	8.4 months	13 months	No acute or late neurological side effects of grade ≥ 2 were reported. No cases of radio-necrosis. Grade 3–4 hematologic toxicity occurred in 3 cases.

KPS, Karnofsky performance status; ECOG, Eastern Cooperative Oncology Group; MRC, Medical Research Council scale; RPA, recursive partitioning analysis; y, years; fx, fractions; TMZ, temozolomide; BEV, bevacizumab; WB, whole brain; SIB, simultaneous integrated boost; NA, not applicable; NS, not specified.

## Data Availability

The data are available upon request from the corresponding author.

## References

[1] Stupp R., Mason W., Van der Bent M., Weller M., Fisher B., Taphoorn M.J., Belanger K., Brandes A.A., Marosi C., Bogdahn U. (2005). European Organisation for Research and Treatment of Cancer Brain Tumor and Radiotherapy Groups, National Cancer Institute of Canada Clinical Trials Group. Radiotherapy plus concomitant and adjuvant Temozolomide for glioblatoma. N. Engl. J. Med..

[2] Walker M.D., Strike T.A. (1976). An evaluation of methyl-CCNU, BCNU and radiotherapy in the treatment of malignant glioma. Proc. Am. Assoc. Cancer Res..

[3] Balducci M., Apicella G., Manfrida S., Mangiola A., Fiorentino A., Azario L., D’Agostino G.R., Frascino V., Dinapoli N., Mantini G. (2010). Single- Arm Phase II Study of Conformal Radiation Therapy and Temozolomide plus fractionated Stereotactic Conformal Boost in High grade Gliomas. Strahlentherapie und Onkologie.

[4] Balducci M., Chiesa S., Diletto B., D’Agostino G.R., Mangiola A., Manfrida S., Mantini G., Albanese A., Fiorentino A., Frascino V. (2012). Low-dose fractionated radiotherapy and concomitant chemotherapy in glioblastoma multiforme with poor prognosis: A feasibility study. Neuro Oncol..

[5] Lee S.W., Fraass B.A., Marsh L.H., Herbort K., Gebarski S.S., Martel M.K., Radany E.H., Lichter A.S., Sandler H.M. (1999). Patterns of failure following high-dose 3-D conformal radiotherapy for high-grade astrocytomas: A quantitative dosimetric study. Int. J. Radiat. Oncol. Biol. Phys..

[6] Mazzola R., Corradini S., Gregucci F., Figlia V., Fiorentino A., Alongi F. (2019). Role of Radiosurgery/Stereotactic Radiotherapy in Oligometastatic Disease: Brain Oligometastases. Front. Oncol..

[7] Mazzola R., Corradini S., Gregucci F., Figlia V., Fiorentino A., Alongi F. (2020). Volume de-escalation in radiation therapy: State of the art and new perspectives. J. Cancer Res. Clin. Oncol..

[8] McAleese J.J., Stenning S.P., Ashley S., Traish D., Hines F., Sardell S., Guerrero D., Brada M. (2003). Hypofractionated radiotherapy for poor prognosis malignant glioma: Matched pair survival analysis with MRC controls. Radiother. Oncol..

[9] Phillips C., Guiney M., Smith J., Hughes P., Narayan K., Quong G. (2003). A randomized trial comparing 35 Gy in ten fractions with 60 Gy in 30 fractions of cerebral irradiation for glioblastoma multiforme and older patients with anaplastic astrocytoma. Radiother. Oncol..

[10] Chang E.L., Yi W., Allen P.K., Levin V.A., Sawaya R.E., Maor M.H. (2003). Hypofractionated radiotherapy for elderly or younger low performance status glioblastoma patients: Outcome and prognostic factors. Int. J. Radiat. Oncol. Biol. Phys..

[11] Roa W., Brasher P.M.A., Bauman G., Anthes M., Bruera E., Chan A., Fisher B., Fulton D., Gulavita S., Hao C. (2004). Abbreviated course of radiation therapy in older patients with glioblastoma multiforme: A prospective randomized clinical trial. J. Clin. Oncol..

[12] Malmström A., Grønberg B.H., Marosi C., Stupp R., Frappaz D., Schultz H., Abacioglu U., Tavelin B., Lhermitte B., Hegi M.E. (2012). Temozolomide versus standard 6-week radiotherapy versus hypofractionated radiotherapy in patients older than 60 years with glioblastoma: The Nordic randomised, phase 3 trial. Lancet Oncol..

[13] Scoccianti S., Krengli M., Marrazzo L., Magrini S.M., Detti B., Fusco V., Pirtoli L., Doino D., Fiorentino A., Masini L. (2018). Hypofractionated radiotherapy with simultaneous integrated boost (SIB) plus temozolomide in good prognosis patients with glioblastoma: A multicenter phase II study by the Brain Study Group of the Italian Association of Radiation Oncology (AIRO). Radiol. Med..

[14] Wick W., Platten M., Meisner C., Felsberg J., Tabatabai G., Simon M., Nikkhah G., Papsdorf K., Steinbach J.P., Sabel M. (2012). Temozolomide chemotherapy alone versus radiotherapy alone for malignant astrocytoma in the elderly: The NOA-08 randomised, phase 3 trial. Lancet Oncol..

[15] Jablonska P.A., Diez-Valle R., Pérez-Larraya J.G., Moreno-Jiménez M., Idoate M.Á., Arbea L., Tejada S., Garcia de Eulate M.R., Ramos L., Arbizu J. (2019). Hypofractionated radiation therapy and temozolomide in patients with glioblastoma and poor prognostic factors. A prospective, single-institution experience. PLoS ONE.

[16] Perry J.R., Laperriere N., O’Callaghan C.J., Brandes A.A., Menten J., Phillips C., Fay M., Nishikawa R., Cairncross J.G., Roa W. (2017). Trial Investigators. Short-Course Radiation plus Temozolomide in Elderly Patients with Glioblastoma. N. Engl. J. Med..

[17] Navarria P., Pessina F., Cozzi L., Tomatis S., Reggiori G., Simonelli M., Santoro A., Clerici E., Franzese C., Carta G. (2019). Phase II study of hypofractionated radiation therapy in elderly patients with newly diagnosed glioblastoma with poor prognosis. Tumori.

[18] Roa W., Kepka L., Kumar N., Sinaika V., Matiello J., Lomidze D., Hentati D., Guedes de Castro D., Dyttus-Cebulok K., Drodge S. (2015). International Atomic Energy Agency Randomized Phase III Study of Radiation Therapy in Elderly and/or Frail Patients With Newly Diagnosed Glioblastoma Multiforme. J. Clin. Oncol..

[19] de Castro D.G., Matiello J., Roa W., Ghosh S., Kepka L., Kumar N., Sinaika V., Lomidze D., Hentati D., Rosenblatt E. (2017). Survival Outcomes With Short-Course Radiation Therapy in Elderly Patients With Glioblastoma: Data From a Randomized Phase 3 Trial. Int. J. Radiat. Oncol. Biol. Phys..

[20] Hulshof M.C., Schimmel E.C., Bosch D.A., González D.G. (2000). Hypofractionation in glioblastoma multiforme. Radiother. Oncol..

[21] Minniti G., De Sanctis V., Muni R., Rasio D., Lanzetta G., Bozzao A., Osti M.F., Salvati M., Valeriani M., Cantore G.P. (2009). Hypofractionated radiotherapy followed by adjuvant chemotherapy with temozolomide in elderly patients with glioblastoma. J. Neurooncol..

[22] Thomas R., James N., Guerrero D., Ashley S., Gregor A., Brada M. (1994). Hypofractionated radiotherapy as palliative treatment in poor prognosis patients with high grade glioma. Radiother. Oncol..

[23] Minniti G., Scaringi C., Lanzetta G., Terrenato I., Esposito V., Arcella A., Pace A., Giangaspero F., Bozzao A., Enrici R.M. (2015). Standard (60 Gy) or short-course (40 Gy) irradiation plus concomitant and adjuvant temozolomide for elderly patients with glioblastoma: A propensity-matched analysis. Int. J. Radiat. Oncol. Biol. Phys..

[24] Pedretti S., Masini L., Turco E., Triggiani L., Krengli M., Meduri B., Pirtoli L., Borghetti P., Pegurri L., Riva N. (2019). Hypofractionated radiation therapy versus chemotherapy with temozolomide in patients affected by RPA class V and VI glioblastoma: A randomized phase II trial. J. Neurooncol..

[25] Zhong L., Chen L., Lv S., Li Q., Chen G., Luo W., Zhou P., Li G. (2019). Efficacy of moderately hypofractionated simultaneous integrated boost intensity-modulated radiotherapy combined with temozolomide for the postoperative treatment of glioblastoma multiforme: A single-institution experience. Radiat Oncol..

[26] Omuro A., Beal K., Gutin P., Karimi S., Correa D.D., Kaley T.J., DeAngelis L.M., Chan T.A., Gavrilovic I.T., Nolan C. (2014). Phase II study of bevacizumab, temozolomide, and hypofractionated stereotactic radiotherapy for newly diagnosed glioblastoma. Clin. Cancer Res..

[27] Bauman S., Gaspar L.E., Fisher B.F., Halperin E.C., Macdonald D.R., Cairncross J.G. (1994). A prospective study of short-course radiotherapy in poor prognosis glioblastoma multiforme. Int. J. Radiat. Oncol. Biol. Phys..

[28] Curran W.J., Scott C.B., Horton J., Nelson J.S., Weinstein A.S., Fischbach A.J., Chang C.H., Rotman M., Asbell S.O., Krisch R.E. (1993). Recursive partitioning analysis of prognostic factors in three Radiation Therapy Oncology Group malignant glioma trials. J. Natl. Cancer Inst..

[29] Therasse P., Arbuck S.G., Eisenhauer E.A., Wanders J., Kaplan R.S., Rubinstein L., Verweij J., Van Glabbeke M., van Oosterom A.T., Christian M.C. (2000). New guidelines to evaluate the response to treatment in solid tumors. European Organization for Research and Treatment of Cancer, National Cancer Institute of the United States, National Cancer Institute of Canada. J. Natl. Cancer Inst..

[30] Fokas E., Wacker U., Gross M.W., Henzel M., Encheva E., Engenhart-Cabillic R. (2009). Hypofractionated stereotactic reirradiation of recurrent glioblastomas: A beneficial treatment option after high-dose radiotherapy?. Strahlenther Onkol..

[31] Navarria P., Minniti G., Clerici E., Tomatis S., Pinzi V., Ciammella P., Galaverni M., Amelio D., Scartoni D., Scoccianti S. (2019). Re-irradiation for recurrent glioma: Outcome evaluation, toxicity and prognostic factors assessment. A multicenter study of the Radiation Oncology Italian Association (AIRO). J. Neurooncol..

[32] Lombardi G., De Salvo G.L., Brandes A.A., Eoli M., Rudà R., Faedi M., Lolli I., Pace A., Daniele B., Pasqualetti F. (2019). Regorafenib compared with lomustine in patients with relapsed glioblastoma (REGOMA): A multicentre, open-label, randomised, controlled, phase 2 trial. Lancet Oncol..

[33] Fiorentino A., Balducci M., De Bonis P., Chiesa S., De Filippo L., Mangiola A., De Rose F., Autorino R., Rinaldi C., Fersino S. (2015). Can elderly patients with newly diagnosed glioblastoma be enrolled in radiochemotherapy trials?. Am. J. Clin. Oncol..

[34] Balducci M., Fiorentino A., De Bonis P., Chiesa S., Manfrida S., D’Agostino G.R., Mantini G., Frascino V., Mattiucci G.C., De Bari B. (2012). Impact of age and co-morbidities in patients with newly diagnosed glioblastoma: A pooled data analysis of three prospective mono-institutional phase II studies. Med. Oncol..

[35] Fiorentino A., Caivano R., Chiumento C., Cozzolino M., Clemente S., Pedicini P., Fusco V. (2012). Comorbidity assessment and adjuvant radiochemotherapy in elderly affected by glioblastoma. Med. Oncol..

